# Unraveling synonymous and deep intronic variants causing aberrant splicing in two genetically undiagnosed epilepsy families

**DOI:** 10.1186/s12920-021-01008-8

**Published:** 2021-06-09

**Authors:** Qiang Li, Yiting Wang, Yijun Pan, Jia Wang, Weishi Yu, Xiaodong Wang

**Affiliations:** 1 Guiyang Maternal and Child Health Care Hospital, Guiyang, 550002 China; 2 Cipher Gene, Ltd., Beijing, 100080 China

**Keywords:** Trio-WES, Aberrant splicing, Synonymous variant, Intronic variant, Undiagnosed rare diseases, Minigene

## Abstract

**Background:**

Variants identified through parent–child trio-WES yield up to 28–55% positive diagnostic rate across a variety of Mendelian disorders, there remain numerous patients who do not receive a genetic diagnosis. Studies showed that some aberrant splicing variants, which are either not readily detectable by WES or could be miss-interpreted by regular detecting pipelines, are highly relevant to human diseases.

**Methods:**

We retrospectively investigated the negative molecular diagnostics through trio-WES for 15 genetically undiagnosed patients whose clinical manifestations were highly suspected to be genetic disorders with well-established genotype–phenotype relationships. We scrutinized the synonymous variants from WES data and Sanger sequenced the suspected intronic region for deep intronic variants. The functional consequences of variants were analyzed by in vitro minigene experiments.

**Results:**

Here, we report two abnormal splicing events, one of which caused exon truncating due to the activation of cryptic splicing site by a synonymous variant; the other caused partial intron retention due to the generation of splicing sites by a deep intronic variant.

**Conclusions:**

We suggest that, despite initial negative genetic test results in clinically highly suspected genetic diseases, the combination of predictive bioinformatics and functional analysis should be considered to unveil the genetic etiology of undiagnosed rare diseases.

**Supplementary Information:**

The online version contains supplementary material available at 10.1186/s12920-021-01008-8.

## Background

Whole-exome sequencing (WES) approach has been highly successful in detecting causative variants in many epilepsy patients as evidenced by its current widespread use in pediatric neurology clinical practice, as well as in large-scale disease-relevant gene discovery studies [1]. Variants identified through parent–child trio-WES yield up to 28–55% positive diagnostic rate across a variety of Mendelian disorders [2, 3]. However, there remain numerous patients who do not receive a genetic diagnosis by exome sequencing indicating that either disease-causing variants could not be caught by inadequate genomic coverage regions or the underlying disease mechanism is still unclear. In some situations, the detected variants are difficult to be classified as pathogenic due to the imperfect bioinformatics pipelines, inaccurate variant interpretations, or insufficient functional evidence, which keep the presumed genetic epilepsy undiagnosed from the clinical practice point of view.

Aberrant splicing has long been known as a major cause of rare genetic disorders [4–6]. Pre-mRNA splicing depends on the precise recognition of consensus exon–intron boundaries and regulatory sequences as well as the interaction of spliceosome snRNP and splicing factors [7]. Any erroneous and inaccurate process may lead to aberrant splicing creating exon skipping, alternative 5′/3′ splicing, intron retention, etc. [8]. Deep intronic variants create cryptic splice donor or acceptor sites, which result in the inclusion of intronic fragment and production of a cryptic exon in mRNA transcript. Functionally, such variants could cause frameshift or introduction of premature termination codon, and subsequently, lead to nonsense-mediated mRNA decay (NMD) or truncated protein [9–11]. Exonic synonymous variants may also affect splicing by introducing a new 5′ or 3′ splice site, activating the cryptic splicing site, or interrupting the exonic splicing enhancers [12, 13]. Deep intronic variants are usually undetectable because they are not covered by WES. Exonic synonymous variants are easily neglected because they are predicted to be unharmful by in silico pipelines.

We retrospectively investigated the negative molecular diagnostics through trio-WES for 15 genetically undiagnosed patients whose clinical manifestations were highly suspected to be genetic disorders with well-defined phenotypes,, such as Dravet syndrome (DS) or genetic epilepsy with febrile seizures plus (GEFS+) and benign familial neonatal epilepsy (BFNE). Genes associated with these epilepsies have been well known. We presumed that the “missing variants” might reside within the gene regions that are not covered by exome sequencing or not revealed by routine variant interrogation pipeline. We scrutinized the rare synonymous variants that may affect splicing from WES data. Moreover, we performed additional experiments to identify deep intronic variants that may be the genetic cause of undiagnosed disease.

## Methods

### Patients

Patients were recruited from pediatric clinics from Guiyang Maternal and Child Health Care Hospital. Trio-WES data from 15 families with initially negative reports collected from July 2019 to June 2020 were re-analyzed. Written informed consents for genetic testing, research, and publication of related data were obtain from all families. Ethical committee of the hospital approved the study with adherence to the Declaration of Helsinki.

### Whole exome sequencing

The exome was captured from peripheral blood DNA using IDT xGen Exome Research Panel (Integrated DNA Technologies, Coralville, Iowa), Subsequent paired-end sequencing was performed by NovaSeq 6000 (Illumina, Santa Clara, California). Reads were aligned to the reference genome GRCh38 with BWA-mem. Genome Analysis Tool Kit (GATK v4) best practices (https://software.broadinstitute.org/gatk/best-practices/) from the Broad Institute was applied for variant calling and, ANNOVAR (http://www.openbioinformatics.org/annovar/) was used for variant annotation including population databases (1000 Genome Project, Exome Variant Server, ExAC, gnomAD, and our in-house population database), published or submitted variants (HGMD, Clinvar) and in-silico pathogenicity predictions for missense variants (SIFT, PolyPhen2, LRT, MutationTaster, FATHMM, CADD, REVEL) and splice site variants (MaxEntScan, NNSplice, dbscSNV). Variants were classified following the guidelines of the American College of Medical Genetics and Genomics (ACMG) [14].

### Sanger sequencing analysis

A highly conserved region in *SCN1A* intron 23 (GRCh38, chr2:166006890–166007890, NM_001165963.2) was amplified using the following primers: Forward-5′- CGCCCTCACCAATCCAGTA -3′, Reverse-5′- AGCTGCGTCAAAGCGTAACT -3′. PCR products were sequenced and analyzed for variant detection. Sanger sequencing was also used for variants confirmation and segregation analysis for family members.

### Minigene construction and splicing analysis

For *KCNQ2*, a splicing reporter of 744 bp fragment containing exon14(106 bp)—intron 14(506 bp)—exon15(132 bp) was generated with wild-type or patient-specific variant (c.1617C > T). For *SCN1A*, a splicing reporter contained pseudo exonA, 1440 bp partial intron 23 including 64-nt conserve region with wild-type or patient-specific variant (c.4002 + 2461T > C) and pseudo exonB. Fragments were synthesized and inserted into Exontrap Vector pcMINI as minigene. Reporters were transfected into HeLa and HEK-293T cells in triplicate and harvested after 48 h. Total RNAs were extracted by Trizol(RNAiso PLUS, TaKaRa, 9109), and cDNAs were then synthesized with 500 ng of input RNA using HifairTM 1st Strand cDNA Synthesis SuperMix for qPCR (gDNA digester plus, YEASEN, 11123ES70). 1 μl of the cDNA mixture was amplified with PrimerSTAR MAX DNA Polymerase (TaKaRa, R045A) and pcMINI vector-expressed exon-specific PCR primers (Forward-5′-TTAACATCTGTGCGTGGATG-3′, Reverse-5′- CGCCCACCAGCTCCACACAC-3′). Amplified products were subjected to run agarose gel and sequencing analysis.

## Result

Two out of 15 families yielded positive results after careful reviews on detailed clinical phenotypes, family history, re-analysis of WES data. Genetic etiologies were identified and confirmed with functional analysis. Clinical features of the rest of 13 families were summarized in Additional file 1: Table S1.

### Clinical findings

#### Family 1

**Fig. 1:**
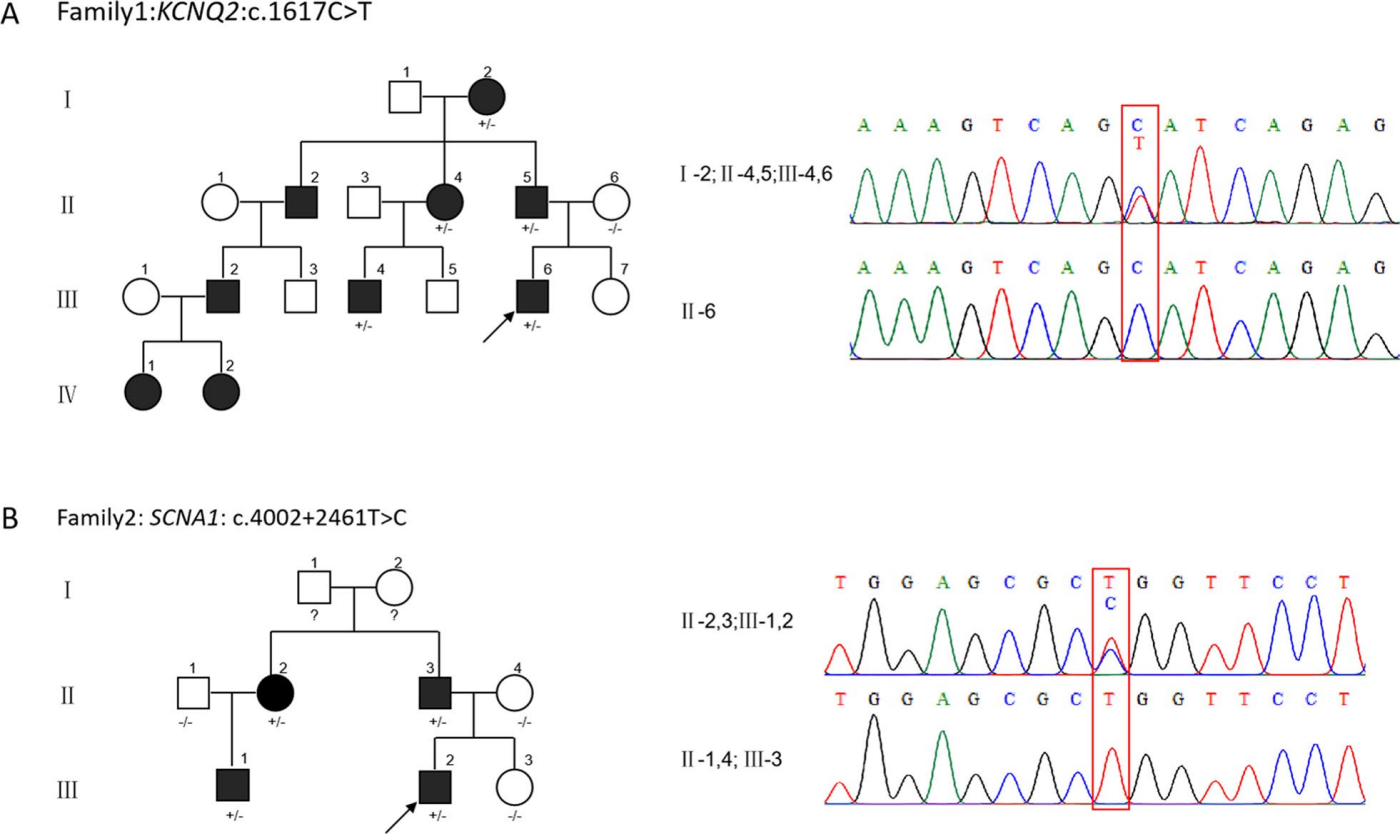
Pedigree and genetic analysis of patient families. **a** Pedigree of family1 exhibited convulsive seizures affected by a synonymous variant c.1617C > T(p.Ser539=) in *KCNQ2*. **b** Pedigree of family2 exhibited febrile or afebrile seizures affected by a deep intronic variant (c.4002 + 2461T > C) in *SCN1A.* Both variants were verified by Sanger sequencing. Proband is indicated by an arrow; Open symbol: unaffected individual; filled symbol: affected individuals; square: male; circle: female; ?: unknown phenotypes; An individual with a heterozygous mutation is indicated by ± , and an individual without a mutation is indicated by -/-

Proband (III-7) from family 1 was a 1-year-old male (Fig. 1a). The first epileptic seizure occurred at 4-month-old. The seizure types were focal to generalized seizures with frequent clustered seizures of short-term. No abnormalities were observed in his perinatal period. Normal developmental milestones were evaluated before and after seizures. Blood biochemistry and cerebrospinal fluid examinations were normal. Brain imaging was normal. No epileptic discharge was detected during video EEG in interictal, and two focal to generalized seizures at onset from the right anterior head were monitored. Low-dose sodium channel blocker Oxcarbazepine (OXC) and Sodium Valproate (VPA) had obvious effects on seizure control (OXC was replaced by VPA due to OXC allergy). The patient became seizure-free for seven months, and VPA was discontinued. The proband had an extensive family history: 9 family members had a history of multiple or frequent seizures from the neonatal period to about 3 months of age, their seizures ceased from a few months to one and a half years old, and their development was normal. The patient was diagnosed as BFNE based on the International League Against Epilepsy (ILAE) classification.

#### Family 2

Proband (II-3) from family 2 was a 3-year-11-month old male (Fig. 1b). He was diagnosed with GEFS + with an extensive family history of febrile seizures plus (FS+). He suffered four times of febrile seizures from 6 months to 2 years old. He turned to afebrile convulsions after 2 years old with tonic–clonic seizures during wake and sleep. The perinatal period was normal. His father (I-3), aunt (I-2), and cousin (II-1) had several febrile seizures before 5 years old, and their development was normal. His language development lagged behind his peers slightly, and his speech was unclear. However, his motor milestones and intellectual development were normal. His blood biochemistry results were normal. His brain MRI and VEEG were unremarkable. He had been treated with Levetiracetam (LEV), which reduced seizures significantly.

### Genetic investigation

#### Identification of synonymous variant in *KCNQ2* in family 1

For patient one, a rare synonymous variant Chr20(GRCh38): g.63414102G > A, NM_172107.3 (*KCNQ2*): c.1617C > T (p.Ser539=)) was identified that was inherited from his father (II-5) (Fig. 1a). This variant was not predicted to affect splicing according to a routine prediction by MaxEntScan, NNSplice, dbscSNV at initial testing. The additional prediction was done by SpliceAI (https://spliceailookup.broadinstitute.org/). It scored 0.93 for donor gain 6 bp upstream and 0.6 for donor loss 14 bp downstream to the variant (pre-mRNA position), which suggested a cryptic splicing donor site that might be activated along with the natural donor site lost. According to ACMG guidelines, this variation was classified as a variant of uncertain significance (VUS). Sanger sequencing results confirmed the same variant carried by the patient’s grandmother (I-2), aunt (II-4), and cousin (III-4) with similar clinical manifestations (Fig. 1a). The variant was co-segregated with the clinical phenotypes within family members.

#### Identification of deep intronic variant in *SCN1A* in family 2

For patient two, no candidate disease-causing variants were detected by the re-analysis of WES data. Referring to the clinical phenotypes of the proband, we decided to focus on ion channel genes *SCN1A*. We amplified the highly conserved region of *SCN1A* intron23 (GRCh38, chr2:166006890–166007890) by specific primers and followed with Sanger sequencing. A variant Chr2(GRCh38): g.166007258A > G, NM_001165963.2 (*SCN1A*): c.4002 + 2461T > C located in a 64 bp of highly conserved DNA region in intron 23 was identified (Fig. 1b). SpliceAI predicted a weak splicing effect as 0.18 for donor-gain 28 bp downstream and 0.18 for acceptor-gain 35 bp upstream to the variant which suggested an intron retention. This variant co-segregated with disease in multiple affected family members (II-2, II-3, III-1) (Fig. 1b).

### In vitro functional analysis

#### *KCNQ2*_c.1617C > T activates a cryptic splice site

**Fig. 2:**
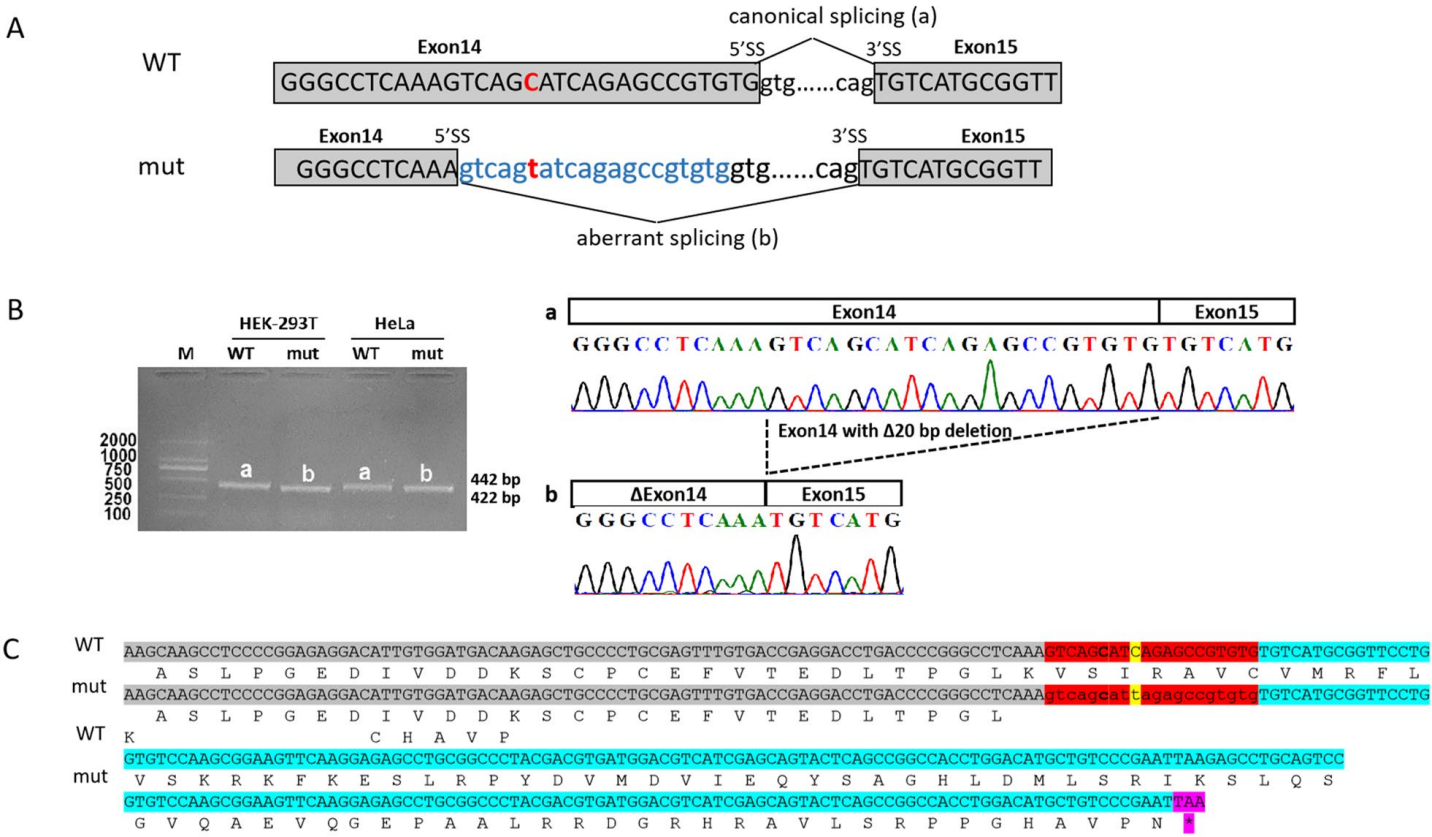
In vitro minigene assay demonstrated an aberrant transcript produced due to the synonymous variant in *KCNQ2*. **a** A schematic illustration of the synonymous variant c.1617C > T (p.Ser539=) (NM_172107.3) (highlighted in red) in *KCNQ2* causing aberrant splicing. **b** RT-PCR amplified minigene transcripts showed the variants cause recruitment of a cryptic splicing donor site in exon 14 at position c.1612 resulted in a 20‐nt deletion. WT, wild‐type; mut: Mutation; 5’ss: donor splice site; 3’ss: acceptor splice site. **c** Deletion of 20-nt in exon 14 caused a frameshift in protein translation and introduced a premature termination codon (PTC) in exon15. Grey highlight: exon14; Cyan highlight: exon15; Red highlight:20-nt deletion. Yellow highlight: variant in this patient; Pink highlight: stop codon (TAA)

Because the patient’s RNA was unavailable, we functionally characterized the synonymous variant using minigene assay. RT-PCR analysis from human cell lines Hella and HEK-293T transfected with mutant construct showed aberrant splicing compared with wild-type. Sequencing of the PCR fragment revealed that the variant resulted in shorter transcripts with 20-nt deletion, owing to the activation of a novel cryptic 5’ donor splice site within exon14 (Fig. 2a, b). The transcript was predicted to produce a prematurely truncated protein (p.Val537Cysfs*39) (Fig. 2c).

#### *SCN1A*_c.4002 + 2461T > C leads to intron retention

**Fig. 3:**
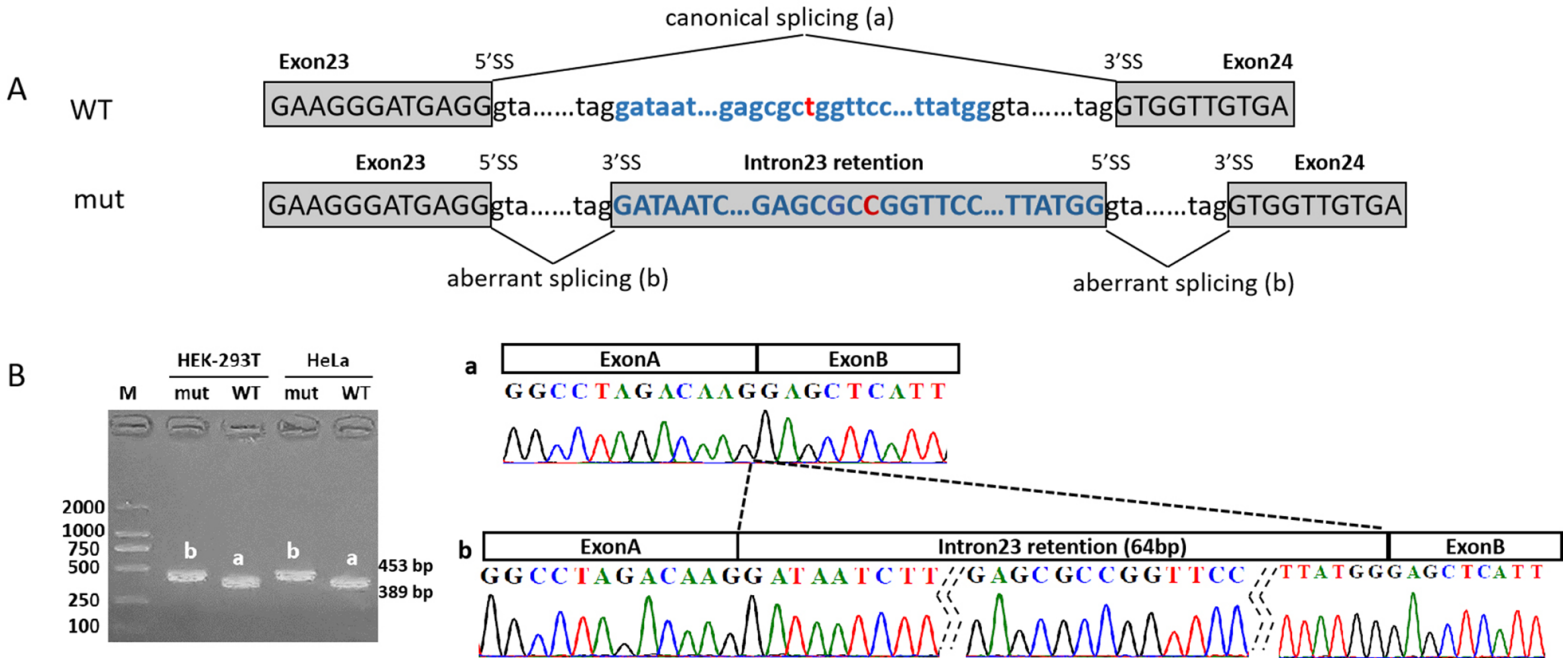
In vitro minigene assay demonstrated an intron retention produced due to the deep intronic variant in *SCN1A*. **a** A schematic illustration of the deep intronic variant c.4002 + 2461T > C (NM_001165963.2) (highlighted in red) in *SCN1A* causing aberrant splicing. **b** RT-PCR amplified minigene transcripts showed the variants activating of cryptic splice sites (3’ss: c.4002 + 2425; 5’ss: c.4002 + 2489) which resulted in a 64-nt intron retention. RT‐PCR: reverse transcription‐polymerase chain reaction; WT, wild‐type; mut: Mutation; 5’ss: donor splice site; 3’ss: acceptor splice site

**Fig. 4:**
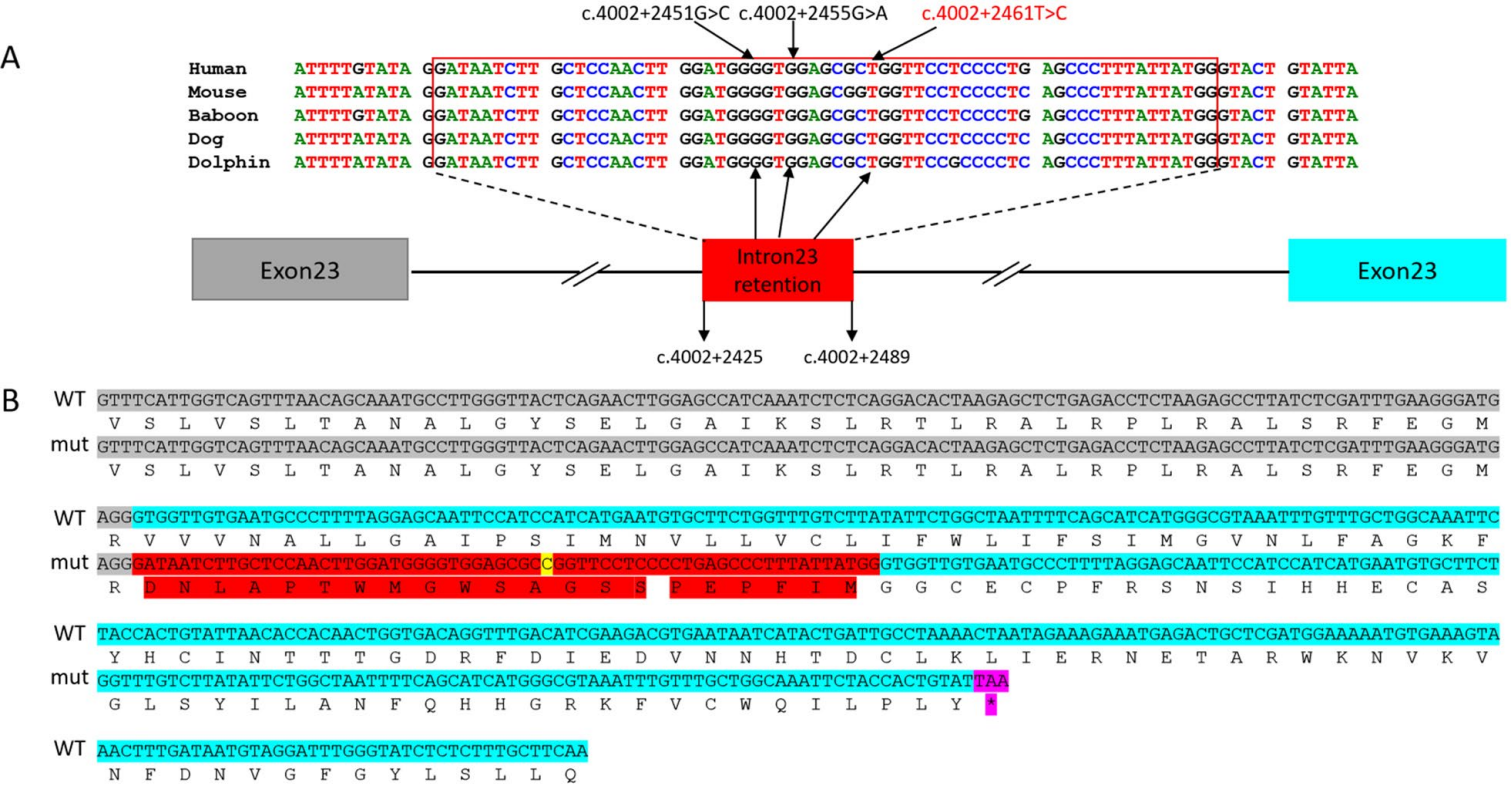
Deep intronic variant c.4002 + 2461T > C in *SCN1A* intron23 is predicted to introduce a premature termination codon (PTC) in exon 24. **a** Alignment of 64-nt intron23 retention showed the sequence conservation across multiple species. Two variants from Carvill et al. are labeled in black and variant identified from this study is labeled in red. **b** The sequences of 64-nt intron 23 retention introduced PTC in exon24 due to frameshift and predictively resulted in NMD or truncated protein. Grey highlight: exon23; Cyan highlight: exon24; Red highlight: 64-nt intron retention. Yellow highlight: variant in this study; Pink highlight: stop codon (TAA)

RT-PCR results showed that minigene products of *SCN1A*_c.4002 + 2461T > C variant were longer than that of wild-type from both Hella and HEK-293T cell lines. Sequencing analysis revealed a 64-nt intron 23 retention, indicating that activation of cryptic splice sites at 3’ss: c.4002 + 2425 and 5’ss: c.4002 + 2489 (Fig. 3). The insertion of 64-nt fragment was predicted to generate a stop codon at amino acid position 65 after the variant (p.Val1294Aspfs*65) (Fig. 4b).

## Discussion

Next-generation sequencing has revolutionized clinical diagnostic testing. However, the sequence information limited to exon and exon–intron boundary regions determines molecular diagnosis rates remain between 28–55% for most diagnostic centers [2, 3]. Disease-causing variations are identified mainly at the genomic level. The effects of variation on mRNA and encoded protein can only be predicted by DNA sequence, very few cases have been experimentally confirmed the effects of variants at both DNA and RNA levels [15]. Current bioinformatics filtering strategies and clinical interpretation guidelines tend to focus on the impact of variants at the amino acid level. This may result in some synonymous variants that affect splicing being filtered out at the early stage of analysis. Similarly, although an increasing data of deep intronic variants are being identified through whole-genome sequencing, such noncoding variants are rarely considered due to the lack of evidence for interpretation [16]. Previous studies suggested that between 9–30% of causative variants in Mendelian disorders cases may act through disruption of splicing [17]. However, only 8.6% (24,976/289,000) of all mutations reported in the Human Gene Mutation Database (HGMD) are splicing mutations (HGMD database, June 2020). Disease-associated splicing variants could be extensively underestimated. Therefore, comprehensive investigations of exonic synonymous and deep intronic variants affecting splicing may be of great benefit to improve the diagnostic yield for patients with rare diseases. In this study, we reported two significant variants that were missed by initial exome testing results in two situations: (1) the splicing variant was not covered by exome sequencing due to its deep intronic location; (2) the synonymous variant was neglected by routine pipeline. We demonstrated that further investigations were necessary to reveal the causes of genetically undiagnosed cases.

It has long been assumed that the effects of synonymous variants on molecular functionality of the genes are minimal, thus synonymous variants are not considered as pathogenic conventionally. However, earlier studies have argued that synonymous variants are as likely to be pathogenic as non-synonymous variants [18], albeit our ability to predict their effects is limited. Synonymous variation *KCNQ2* c.1617C > T (p.Ser539=) identified in family 1 via WES was left out in our first round of variant detection because the variant did not meet the protein-coding nonsynonymous variant filtering criteria. Also, none of our splicing-altering prediction algorithms gave high specific scores. We deployed the newer machine learning-based approach SpliceAI [19]. It predicted a cryptic splicing donor site 6-bp upstream of the variant activated with a high score. In vitro minigene assay demonstrated that 20-bp of exon 14 containing the synonymous variant (p.Ser539=) was skipped in the mRNAs leading to an unexpected frameshift. Consistent with previous studies [16, 20], our result suggests that SpliceAI, outperformed other splicing prediction algorithms, could assist in the clinical interpretation of potential splicing-altering variants.

Data analysis of mRNA and whole genome sequencing showed that pathogenic variations can occur in deep introns for over 75 disease-associated genes [11]. The most common deep intronic variation is that the activation of non-canonical splicing sites or the change of splicing regulatory elements leading to the generation of pseudo exons. In this study, we identified a deep intronic variation of *SCN1A*(c.4002 + 2461T > C) that occurred at a very conserved region across multiple species (Fig. 4a). This variation activates the non-canonical splicing sites and leads to 64-nt retention of intron 23 (c. 4002 + 2425 to c. 4002 + 2489). Carvill reported two epilepsy-associated variants that were located in the same region (Fig. 4a) [21]. Similar to our variant c.4002 + 2461T > C, pathogenic variant c.4002 + 2451G > C was identified in a Dravet patient. This variant was predicted to activate cryptic donor/acceptor sites causing aberrant splicing to include a 64-nt fragment of intron 23 to be transcribed into pre-mRNA. Once transcribed, a premature termination codon (PTC) was predicted to be generated at the position of 43rd amino acid in exon 24 (Fig. 4b). Another pathogenic variant c.4002 + 2455G > A identified in a patient with FS + generated a stop codon lying in the 64-nt intronic fragment. This exon that resulted in nonproductive transcripts via alternative splicing was also called ‘poison exon’ (PE). A nonsense variant within this exon or an out-of-frame aberrant transcript will trigger NMD or produce a truncated form of SCN1A protein, therefore reduce the amount of full-length SCN1A protein, and cause epilepsy due to haploinsufficiency [22]. Our data support that aberrant PE inclusion could be the underlying mechanism for some unsolved genetic epilepsies. Current panel or WES that are widely used in clinical practice are not capable of capture causative genetic variants in the deep intronic region including putative poison exons, therefore, we suggest that additional sequencing of the targeted area and confirmatory assay should be considered.

Genetic changes underlying some epilepsies with the well-established gene-phenotype association are well understood. Proband from family 1 clinically displayed BFNE, which is a genetic epilepsy syndrome characterized by clustered, recurrent newborn or infantile seizures with positive family history. Patients usually manifest seizures around a few days or months of life but with normal interictal EEG, MRI, developmental and intellectual outcomes. Pathogenic variants in *KCNQ2* gene are known to be a common cause of the disease [23–27]. Studies suggested that drugs acting on sodium channels should be considered as first-line treatment in patients with *KCNQ2*-associated epilepsy [26–29]. Based on clinical diagnosis proband from family 1 was treated with a low dosage of sodium channel blocker OXC and VPA, and seizures were controlled effectively. It is also worth noting that *PRRT2* is another pathogenic gene associated with benign epilepsy with infantile onset and usually comes into consideration first for patients who clinically manifest focal seizure clusters and unremarkable interictal EEG and MRI findings. Identification of *KCNQ2* pathogenic variants is significant for early precision treatment and prognosis management.

Proband from family 2 with a positive family history was clinically diagnosed as GEFS+, which is a familial epilepsy syndrome characterized by clinical phenotypes ranging from mild febrile seizures, FS+ to less commonly afebrile seizures with atonic, myoclonic, or absences seizures. *SCN1A* is the most significant gene associated with the broader spectrum of GEFS+ [22, 30–33]. Notably, it has been well shown the potential pitfalls of Sanger sequencing to reveal *SCN1A* pathogenic variants in mutation-negative DS patients, both due to technical limitations and human errors [34]. Our case further demonstrated that for cases where *SCN1A* is highly suspected and no exome diagnosis is made, supplemental approaches need to be considered to discover the possible cause of the associated disease.

WES of both cases returned with negative results initially, however, clinical evidence and phenotype/genotype co-segregation strongly suggested genetic etiology underlined. Our study confirmed the pathogenicity of the synonymous variant of *KCNQ2* and the deep intronic variant of *SCN1A* in two families and indicated that certain consequences of DNA variants could only be evaluated at the RNA transcript level. Thus, for some undiagnosed, highly suspected genetic conditions, we recommend RNA level tests in the context of phenotypic information to be performed to increase diagnostic rates.

## Conclusion

In summary, we present two genetically undiagnosed cases caused by synonymous and deep intronic variants that were initially unsolved by WES. With genetic and functional evidence, we reveal that both variants generate aberrant splicing, which could lead to NMD or truncated protein, therefore, cause disease-associated clinical manifestations. Negative WES results of clinical presumed genetic cases with the well-established gene-disease association and positive family history should provoke re-examination, and particular attention should be drawn to intronic or synonymous variants that are usually overlooked. We suggest that the combination of predictive bioinformatics and functional analysis should be used to unveil the genetic etiology of undiagnosed genetic diseases.

## Supplementary Information

**Additional file 1**. Clinical features of 13 families that are investigated in this study.

## Data Availability

The datasets generated during the current study are available in the ClinVar (http://www.ncbi.nlm.nih.gov/clinvar/) repository, and can be found under the accession number VCV001077142.1, and VCV001077141.1, for *KCNQ2*, and *SCN1A*, respectively.

## Abbreviations

WES: Whole-exome sequencing
RT‐PCR: Reverse transcription‐polymerase chain reaction
NMD: Nonsense-mediated mRNA decay
DS: Dravet syndrome
GEFS+: Genetic epilepsy with febrile seizures plus
BFNE: Benign familial neonatal epilepsy
OXC: Oxcarbazepine
LEV: Levetiracetam
VPA: Valproate
